# Association Between Activity Diversity and Frailty in Community-Dwelling Older Japanese: A Cross-Sectional Study

**DOI:** 10.1093/geroni/igaa057.600

**Published:** 2020-12-16

**Authors:** Junta Takahashi, Shuichi Obuchi, Hisashi Kawai, Kaori Ishii, Koichiro Oka, Yoshinori Fujiwara, Hirohiko Hirano, Kazushige Ihara

**Affiliations:** 1 Tokyo Metropolitan Institute of Gerontology, Itabashi-Ku, Tokyo, Japan; 2 Waseda University, Tokorozawa, Saitama, Japan; 3 Tokyo Metropolitan Institute of Gerontology, Tokyo, Japan; 4 Hirosaki University, Itabashi-Ku, Tokyo, Japan

## Abstract

In addition to intensity and quality, diversity of activity will be an important factor to explain health outcomes among older adults. Few studies, though, examined an association between activity diversity and health outcomes. This study aimed to examine the association between activity diversity and frailty among community-dwelling older Japanese. Participants were community-dwelling older adults who participated in a cohort study, the “Otassya-Kenshin” in 2018 . The participants were asked frequency of 20 daily activities, inside/outside chores, leisure activities with/without physically, direct/indirect contact with friends and so on, in a week and activity diversity score were calculated using the formula of Shannon’s entropy. Frailty was defined by the Japanese version of the Cardiovascular Health Study criteria. The difference in diversity score between frail and non frail were examined by t-test. Logistic regression analysis with covariates, age, sex, economic status, living alone, BMI, Mini-Mental State Examination, and IADL was adopted to find association between activity diversity score and presence of frailty. Of 652 participants (age: 72.8±6.3, women: 60.6%) analyzed, 27 (4.1%) were defined as frail. Frailty group revealed significantly lower activity diversity score than non-frailty group (0.66±0.11 vs 0.75±0.08, P<0.01). 0.2 point of decrease in diversity score increase 5 times chance of frailty after controlling covariates. We found significant relationship between activity diversity and health outcome among older subjects. The activity diversity may provide additional information to number or intensity of activity.

